# Telomere-Related Disorders in Fetal Membranes Associated With Birth and Adverse Pregnancy Outcomes

**DOI:** 10.3389/fphys.2020.561771

**Published:** 2020-10-02

**Authors:** Jossimara Polettini, Marcia Guimarães da Silva

**Affiliations:** ^1^Universidade Federal da Fronteira Sul (UFFS), Programa de Pós Graduação em Ciências Biomédicas, Faculdade de Medicina, Campus Passo Fundo, Brazil; ^2^Universidade Estadual Paulista (UNESP), Faculdade de Medicina, Departamento de Patologia, Botucatu, Brazil

**Keywords:** telomere shortening, oxidative stress, parturition, gestation, prematurity, membrane premature rupture

## Abstract

Telomere disorders have been associated with aging-related diseases, including diabetes, vascular, and neurodegenerative diseases. The main consequence of altered telomere is the induction of the state of irreversible cell cycle arrest. Though several mechanisms responsible for the activation of senescence have been identified, it is still unclear how a cell is indeed induced to become irreversibly arrested. Most tissues in the body will experience senescence throughout its lifespan, but intrinsic and extrinsic stressors, such as chemicals, pollution, oxidative stress (OS), and inflammation accelerate the process. Pregnancy is a state of OS, as the higher metabolic demand of the growing fetus results in increased reactive oxygen species production. As a temporary organ in the mother, senescence in fetal membranes and placenta is expected and linked to term parturition (>37 weeks of gestation). However, a persistent, overwhelming, or premature OS affects placental antioxidant capacity, with consequent accumulation of OS causing damage to lipids, proteins, and DNA in the placental tissues. Therefore, senescence and its main inducer, telomere length (TL) reduction, have been associated with pregnancy complications, including stillbirth, preeclampsia, intrauterine growth restriction, and prematurity. Fetal membranes have a notable role in preterm births, which continue to be a major health issue associated with increased risk of neo and perinatal adverse outcomes and/or predisposition to disease in later life; however, the ability to mediate a delay in parturition during such cases is limited, because the pathophysiology of preterm births and physiological mechanisms of term births are not yet fully elucidated. Here, we review the current knowledge regarding the regulation of telomere-related senescence mechanisms in fetal membranes, highlighting the role of inflammation, methylation, and telomerase activity. Moreover, we present the evidences of TL reduction and senescence in gestational tissues by the time of term parturition. In conclusion, we verified that telomere regulation in fetal membranes requires a more complete understanding, in order to support the development of successful effective interventions of the molecular mechanisms that triggers parturition, including telomere signals, which may vary throughout placental tissues.

## Introduction

Telomeres are a highly conserved system that plays a central role in maintaining the integrity of the genome and cell. In somatic cells, telomeres reach a critical short length over the lifespan, or under the influence of stressors. Therefore, telomere length (TL) is an important feature of cell aging or senescence, which suggests the idea of a “biological clock,” or a marker of cell replication (Hayflick, 1965; Blackburn, 2001).

In pregnancy, senescence has been related to term delivery mechanisms (Menon et al., 2014a; Behnia et al., 2015; Gomez-Lopez et al., 2017). The concept of placental cell aging in term delivery was first proposed in the 1970s; however, only lately has a relatively large number of studies examined the relationship between cell senescence and the consequent morphological changes in pregnancy. Placenta and fetal membranes constitute temporary tissues in the maternal body, and therefore, are “aged” and ready to be eliminated by the time of the term neonate is born. Thus, cellular senescence may be related to gestational complications if the process is activated prematurely (Behnia et al., 2015; Menon, 2016; Cox and Redman, 2017; Arias-Sosa, 2018). One of the main characteristics of senescent cells is the production of inflammatory cytokines, which indicates a possible role of cellular senescence as an effector pathway that converges to trigger parturition. Intrinsic and extrinsic stressors that induce variation in prenatal exposures and maternal states and conditions, such as cigarette smoking, air pollution, diabetes, obesity, oxidative stress, and inflammation are associated with cellular aging, evidenced by the shortening of telomeres in fetal cells (Whiteman et al., 2017). Thus, characteristics of maternal health status and behaviors during pregnancy may influence the individual’s susceptibility or propensity to disease in later life. However, few studies have shown a direct correlation between telomere dysfunction specifically in fetal membranes and fetal or developmental programming.

Thus, we begin this review with a brief overview of telomere structure and functions, followed by a description of telomere-related senescence mechanisms and the role of telomere dynamics in pregnancy. We then proceed to discuss the findings regarding fetal membranes telomere-senescence-mediated parturition and adverse gestational outcomes. We conclude by summarizing current knowledge blanks and future research directions.

## Telomeres: Structure and Functions

**Figure 1:**
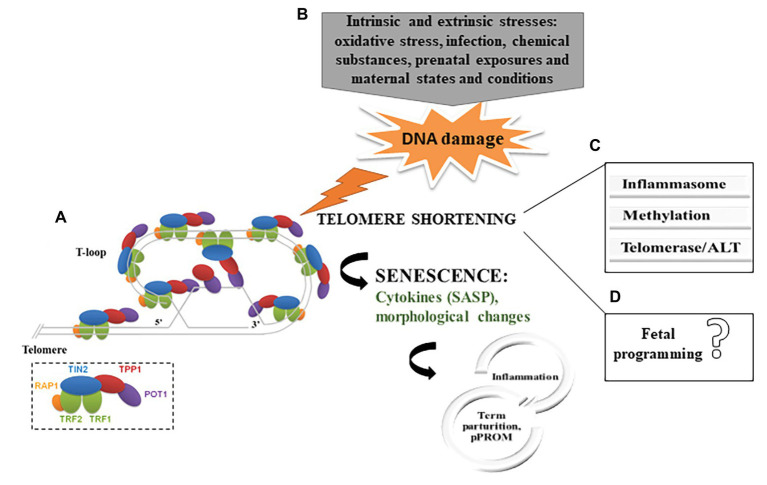
**(A)** Telomere structure and telomere-binding proteins. The six shelterin proteins are depicted in the small box (Adapted from Lu et al., 2013). **(B)** Summary of the proposed mechanism for telomere shortening in fetal membranes in parturition. **(C)** Potential mechanisms of telomere-senescence-mediated parturition and adverse gestational outcomes in fetal membranes. **(D)** No evidence that telomeres dysfunction in fetal membranes has a direct effect on fetal programing (SASP, senescence-associated secretory phenotypes; ALT, alternative lengthening of telomeres; and pPROM, preterm premature rupture of membranes).

Telomeres are nucleoprotein structures at the end of chromosomes that play a vital role in maintaining genomic stability and protecting the chromosomes against fusion and degradation (Blackburn, 1991; Blackburn et al., 2015). In humans, telomeres consist of 2–20 kb of non-coding double-stranded DNA formed by a conserved hexameric (TTAGGG) tandem repeat DNA sequence and a 3' overhang of the G-rich strand, which folds back into the double-stranded DNA, forming a structure known as the *t*-loop, important for protecting the genome from nucleolytic degradation, unnecessary recombination, repair, and interchromosomal fusion (Lu et al., 2013; Blackburn et al., 2015). Besides having a unique DNA sequence, the protection of chromosomes depends on the association and interaction of human telomeres with the shelterin complex, which contains six specific proteins (TRF1, TRF2, POT1, TIN2, TPP1, and RAP1). This network provides a compact chromatin structure that limits the accessibility of DNA damage repair (DDR) machinery and decreases its mistaken recognition at the telomere region (Bandaria et al., 2016; Fathi et al., 2019) (Figure 1A). Since DNA polymerase is unable to fully replicate the 3' end of the DNA strand, telomeres lose part of its sequence with each cell division and reach a critical short length, which, in turn, leads to cellular senescence (Stewart et al., 2003).

Stem cell compartments and embryonic stem cells present telomerase activity; this is a ribonucleoprotein complex, composed of telomerase reverse transcriptase (TERT) and rRNA telomerase component (TERC) subunits and serves as a template for the addition of telomeric repeats to chromosome ends. However, TERT expression and telomerase activity are often very low or undetectable in somatic cells, which explain, in part, the limited capacity of somatic cells to replicate (Rubtsova et al., 2012). Other pathways, such as the alternative lengthening of telomeres (ALT), have been reported in cancer cells (De Vitis et al., 2018). ALT is still not a well-known process, but it is related to telomeric recombination and may be activated when telomerase is repressed. Nonetheless, cultured cells over passages show limited replication capacity, which has been attributed mainly to the shortening of telomeres (Burton and Krizhanovsky, 2014).

Cell aging is a physiological process, as telomeres undergo steady attrition during the proliferation of normal cells; this can be either beneficial or detrimental to the organism. On one hand, it contributes to tumor suppression, limiting tissue damage, and possibly embryonic development, while on the other, it may be associated with aging-related diseases, impaired tissue regeneration, and cellular dysfunction, as well as pregnancy complications (Howcroft et al., 2013; Burton and Krizhanovsky, 2014; Menon et al., 2016; Arias-Sosa, 2018).

## Telomere-Related Senescence Mechanisms

*Replicative senescence* is characterized by cellular proliferative capacity, which depends ultimately on progressive telomere shortening to a critically short length, responsible for the limited number of cell divisions (Bekaert et al., 2005). Therefore, TL is one the main inducers of cell aging or senescence, which suggests the idea of a “biological clock,” or “Hayflick limit,” first described in human fibroblasts cultured *ex vivo* as a proliferative limitation on cells, despite their viability (Hayflick, 1965; Campisi and D’Adda Di Fagagna, 2007; Xu et al., 2013).

The main telomere-related senescence mechanisms include DNA structure dysfunction and modifications. Chromatin and histones structure dysfunction is a key point associated with telomere shortening, which can be triggered by diverse pathways. First, a decrease in histone levels has been observed in human senescent fibroblasts compared to levels in younger cells, impairing processes such as replication, transcription, and DNA repair (O’Sullivan et al., 2010; Adams et al., 2013). Moreover, the majority of lysines on histones are physiologically hypoacetylated near telomeres, contributing to the genomic silencing of this region; thus, any histone modifications, such as acetylation, may interfere with the *t*-loop telomere structure. Such modification may also repress hTERT expression in human cells, such that senescence signaling is initiated (Cong and Bacchetti, 2000). Equally, changes in the structural and epigenetic integrity of telomeres throughout population doubling have an impact on core histones and their chaperones, which, in turn, ultimately lead to senescence (O’Sullivan et al., 2010).

Additionally, chromatin structure is also determined by DNA methylation and, although telomere sequence do not contain genes or CpG sequences, the subtelomeres regions (transition regions between the terminal telomeric repeats and the chromosome-specific regions) are notably CpG-rich, therefore, more prone to be physiologically highly methylated (Toubiana and Selig, 2020). Previous studies have shown that aberrant methylation of subtelomeric DNA exists in many diseases, and it has an impact on the TL regulation, as shorter telomeres are significantly associated with decreased methylation levels at most of CpG sites (Buxton et al., 2014; Hu et al., 2019). Changes in the methylation status of different CpG sites are typical in cancer cells (Joyce et al., 2018), but they have also been observed in senescent cells. This has been used by researchers to successfully predict the age of several different tissues and predispositions to aging-related diseases (Bell et al., 2019). This tool using a set of CpG sites is named epigenetic clock, which starts during development when fetal tissues, embryonic, and induced pluripotent stem cells reveal a DNA methylation age (DNAm age) (Horvath, 2013; Bell et al., 2019).

Besides replicative senescence, *stress-induced senescence* demonstrates that the Hayflick limit is no longer a constant but can vary depending on influencers of telomere loss, such as oxidative damage and/or decrease in antioxidative defense. Thus, the mechanisms described above can be influenced by stressors and accelerate the cell aging process. The accumulation of intrinsic and extrinsic stresses is a well-known pathway that triggers telomere dysfunction and impairs telomere end replication (Pickett and Reddel, 2012; Tan and Lan, 2017), mainly through oxidative stress (OS) (von Zglinicki, 2002; Tan and Lan, 2017).

Under conditions of genomic stability, DNA damage activates DDR complex that coordinates repair and cell cycle progression. Since the telomere is a guanine-rich region (triple structure), it is more vulnerable to OS damage compared to the general genome (von Zglinicki et al., 2000; Stewart et al., 2003; Xu et al., 2013); therefore, telomere dysfunction ultimately leads to cell cycle arrest (Rossiello et al., 2014). In mammalian cells, there are two main DDR mechanisms that address double strand breaks (DSBs): homologous recombination (HR) and non-homologous end joining (NHEJ); the latter is related to telomeric DNA. It has been suggested that NHEJ is inhibited by TRF2 shelterin, preventing chromosomal fusions, and, therefore, end-to-end fusion. Conversely, NHEJ is also the main mechanism for DNA ligase *N*-dependent chromosomal fusions that occur between uncapped telomeres, which suggests a selective regulatory switch, from preventing recombination to promoting it (Evans and Cooke, 2007). OS is known to accelerate telomere attrition *in vitro* and *in vivo*. Pineda-Pampliega et al. (2020) described how oxidative stress shortens telomeres in free-living white stork chicks. According to the authors, the administration of antioxidants had a functional effect on oxidative stress. Furthermore, environmental and behavioral stressors were found to induce OS-induced telomere damage. Exposure to different organic pollutants such as dioxins, furans, and polychlorinated biphenyls (PCBs) through food, water, and air, which occur during the human lifetime, may change TL in peripheral blood leukocytes (Shin et al., 2010; Mitro et al., 2016; Karimi et al., 2020). Serum levels of organochlorine pesticides can be associated with oxidative stress and systemic inflammation that lead to telomere shortening (Karimi et al., 2020). The cellular exposure to non-ortho PCBs and toxic equivalency was associated with increased leukocyte TL in a study population of American adults, contributing population-level findings to the evidence that exposure to environmental contaminants may influence telomere regulation (Mitro et al., 2016). In this same direction, Shin et al. (2010) analyzed the impact of low-dose exposure to persistent pollutants, i.e., lipophilic xenobiotics, on the TL of peripheral blood leukocytes in healthy persons. It was concluded that TL increases with low doses of exposure, suggesting that low doses may act as tumor promoters in carcinogenesis in humans.

**Figure 2:**
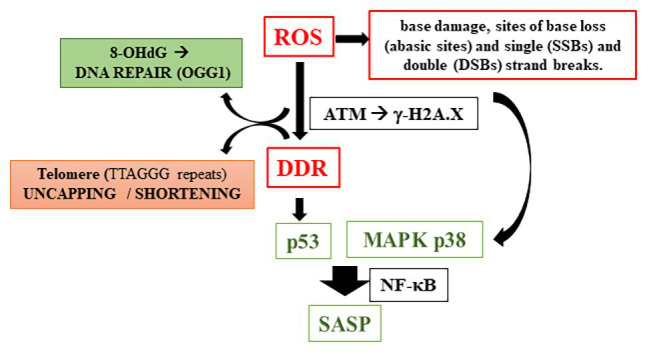
Reactive oxygen species induction of telomere shortening (8-OHdG, 8-hydroxy-2'-deoxyguanosine; OGG1, 8-oxoG-DNA glycosylase; ATM, ataxia telangiectasia mutated kinase; DDR, DNA damage response; MAPK, mitogen-activated protein kinases; and SASP, senescence-associated secretory phenotypes).

Therefore, how does oxidative stress cause telomere shortening? Although there are many suggested pathways for answering this question, the increase of reactive oxygen species and/or decrease in the antioxidant capacity mainly lead to damage in cellular structures, mostly inducing oxidized base in the DNA and consequent DDR defects. Kawanishi and Oikawa (2004) have reported DNA damage caused by the treatment of fibroblast with UVA irradiation, including 8-hydroxy-2'-deoxyguanosine (8-OHdG) formation, specifically at the GGG sequence in the telomere sequence, which was correlated with a decreased in TL. The enzyme responsible for repairing this DNA damage is an 8-oxoG-DNA glycosylase (OGG1), and its action begins by excising the damaged base and subsequent replacement of the modified base (Rosenquist et al., 1997). This local DNA damage can disrupt cell replication; consequently, if the repair mechanism by OGG1 is impaired, a damage to single-stranded DNA strand in the telomere region occurs, which contributes to its shortening. Under the repair failure, mechanisms of DDR by sensor proteins such as the ataxia telangiectasia mutated (ATM) kinase is activated, which regulates the early step(s) of DNA damage signaling, and thereby controls DDR. Persistent DNA damage in response to overwhelming OS causes DNA breaks followed by the phosphorylation of the histone H2AX (γ-H2AX). This *via* induces mechanism of cellular damage involving p53 activation, as well described in cells such as fibroblasts. Alternatively, the route that leads to senescence can be p53-independent, through the activation of mitogen-activated protein kinases (MAPKs) pathway (Iwasa et al., 2003). Salminen et al. (2012) have reported either pathway converges to a downstream activation of NF-κB signaling. In turn, NF-κB system is linked to inflammatory responses in cellular senescence. It is important to note that senescent cells acquire many changes in gene expression, resulting in changes in secreted proteins, such as growth factors, proteases, chemokines, and cytokines, that, together, characterizes the senescence-associated secretory phenotype (SASP; Davalos et al., 2010; Freund et al., 2010; Rodier and Campisi, 2011) (Figure 2).

Additionally, a newly discovered telomere-related stressor is sex hormone concentrations, but a direct relation between sex hormones and TL, if any, remains uncertain. Preliminary evidence suggests that sex steroid hormones could be involved in enhancing telomerase activity since serum dihydrotestosterone and estradiol are positively correlated with leukocyte TL independently of age (Yeap et al., 2016, 2020). However, other studies have demonstrated no association of short TL with sex hormones in healthy men and women (Coburn et al., 2018; Gu et al., 2020).

As a result of telomere-related OS disorders, some pathological conditions have been described, including diabetes and vascular disease (Blackburn et al., 2015). Primary cultures of fibroblasts were used to examine the impact of a diabetic environment on telomeres and, under elevated glucose conditions, relative TL loss was observed in this model (Sutanto et al., 2019). On the other hand, type 2 diabetes mellitus patients with non-alcoholic fatty liver disease have a significantly longer leukocyte TL than patients without non-alcoholic fatty liver disease (Zhang et al., 2019). Telomere shortening has been associated with premature vascular aging, which may be involved in lower-extremity amputation in patients with type 1 diabetes at high vascular risk (Sanchez et al., 2020). Of particular interest here, telomere dysfunctions are associated with placental aging in the etiology of parturition and adverse pregnancy outcomes, in which fetal membranes and gestational tissue play a crucial role.

## Fetal Membranes Telomere-Senescence in Parturition and Adverse Gestational Outcomes

In recent years, new evidences have shown that cell senescence is related to term delivery mechanisms (Menon et al., 2014b; Behnia et al., 2015; Gomez-Lopez et al., 2017). The concept of placental cell aging in term delivery was first proposed in the 1970s (Rosso, 1976); however, until recently few studies had reported on cellular senescence related to oxidative stress and its consequent morphological changes (Menon et al., 2012; Polettini et al., 2015b). This process is believed to be physiological since the placenta and fetal membranes constitute temporary tissues in the maternal body, and therefore, would be “aged” and ready to be eliminated after the birth of the neonate (Behnia et al., 2015; Menon, 2016; Cox and Redman, 2017; Arias-Sosa, 2018).

Recent investigations have revealed that term pregnancies are characterized by increased OS that induces DNA damage (Menon, 2016; Cox and Redman, 2017; Arias-Sosa, 2018); therefore, TL is affected. Gestational tissues show evidence of senescence, given that the telomere attrition rate is negatively correlated with gestational age, thus the closer to term, the shorter the telomeres in fetal and placental cells (Gielen et al., 2014; Casavant et al., 2019). Accordingly, recent findings have demonstrated that fetal membranes from term in labor pregnancies had shorter TL than both preterm and term not in labor pregnancies, suggesting the senescence of term placentas along with labor (Menon et al., 2012; Polettini et al., 2015a; Colatto et al., 2020). The same results were observed in fetal membranes and placenta in mouse (Phillippe et al., 2019), providing support for the hypothesis that shorter telomeres at term potentially function as a biologic clock for parturition.

Besides TL analysis, term labor tissues show features and markers of senescence, such as histological enlarged cells and organelles, granulated nuclei, and more intense staining of senescence-associated β-galactosidase (a lysosomal enzyme; Menon et al., 2014a; Behnia et al., 2015) that strengthens the indication of fetal membranes senescence by parturition time. Among the changes in senescent cells, the production of inflammatory mediators is of particular interest (Freund et al., 2010), as they induce parturition. Fetal membranes play a crucial role in this process, as these tissues are in close contact with the amniotic fluid and, consequently, the fetus (Parry and Strauss, 1998). In the third trimester of pregnancy, chorioamniotic cells increase their production of mediators, especially pro-inflammatory cytokines, immunomodulatory cytokines, neutrophil recruitment chemokines, and arachidonic acid metabolites (Kamel, 2010; Hua et al., 2013; Romero et al., 2018). These mediators are essential to stimulate the production of prostaglandins and consequent uterine contractility (Romero et al., 2006).

Therefore, as is well-known, inflammation is a key point in parturition, even in the absence of intrauterine infection, and, in such cases, inflammation may come from the senescence of fetal membranes (Behnia et al., 2016; Martin et al., 2017). Behnia et al. (2015) found higher concentrations of pro-inflammatory SASP markers (granulocyte macrophage colony-stimulating factor and interleukin-6 and -8) in the amniotic fluid of women in labor at term than in women not in labor. Additionally, bioinformatics analysis has shown, under both term and preterm conditions, that maternal exosomes (30–150 nm particles that propagate to distant sites) carry proteins associated with inflammatory and metabolic signaling (Menon et al., 2019). Besides cellular alterations to the pro-inflammatory profile, the inflammasome might be activated during the parturition process by senescent cells with shortened telomeres. Recent findings have revealed telomere dysfunction as a cause of macrophage mitochondrial abnormalities, OS and hyperactivation of the NOD-like receptor family pyrin domain-containing protein 3 (NLRP3) inflammasome (Kang et al., 2018). Likewise, fetal membranes from women who underwent term labor had higher concentrations of NLRP3 (Romero et al., 2018); thus, dysfunctional telomeres might work as a primary factor and cooperate to amplify inflammasome signaling related to parturition signaling. Crosstalk between these pathways may prove to be a key molecular mechanism of immunosenescence that has been reviewed elsewhere (Jose et al., 2017; Ventura et al., 2017) (Figure 1B).

In this context, telomere-related cellular aging may be linked to gestational complications if the process is activated prematurely (Biron-Shental et al., 2010; Menon et al., 2012; Smith et al., 2013). Adverse pregnancy outcomes, such as stillbirth, intra uterine growth restriction, and preeclampsia are related to trophoblast dysfunction and attributed to placental villous telomere shortening (Biron-Shental et al., 2014; Ferrari et al., 2016; Paules et al., 2019). As mainly of these adversities are linked to placental dysfunction, the role of senescence in fetal membranes has been poorly investigated in these complications.

One of the firsts reports in fetal membranes demonstrated shorter TL from preterm premature rupture of membranes (pPROM) compared to preterm labor (PTL) with intact membranes pregnancies at the same gestational age, and the first was similar to term (Menon et al., 2012). More recently, structural and histological changes in fetal membranes from women undergoing pPPROM were found to be compatible with senescence, suggesting its role in disrupting membranes remodeling and homeostasis, with overwhelming ROS-associated inflammation (Menon et al., 2014a; Behnia et al., 2015; Menon, 2016; Menon and Richardson, 2017). Mechanistically, telomere shortening in pPROM is likely a result of senescence activators, such as MAPKs, that are increasingly expressed in fetal membranes in pregnant women with pPROM (Lappas et al., 2011). Dutta et al. (2016) have reinforced that prosenescence stress kinase (p38MAPK) activation, OS damage, and signs of senescence are pronounced in fetal membranes from pPROM in comparison with PTL with no rupture of membranes. Accordingly, the induction of OS caused significant protein peroxidation in the amniotic sac in mouse, which was associated with p38MAPK activation and senescence, in addition to increased concentrations of pro-inflammatory cytokines in amniotic fluid (Polettini et al., 2018). Commonly, OS activates a specific p53 transcriptional response in diverse tissues, which regulates the cellular response to DNA damage; however, fetal membranes fail in activating p53 under OS, suggesting a diverse pathway triggering senescence in this tissue (Polettini et al., 2015a) (Figure 1B).

Important to note that stressed and injured cells and tissue release stimulatory molecules, such as host-derived damage-associated molecular patterns (DAMPs), that signaling through toll-like receptors (TLRs) and activates cellular response. Therefore, telomere shortening in fetal membranes may also provide additional signs to initiate parturition. In senescent fetal membranes, two DAMPs have been reported, the high mobility group box 1 (HMGB1) and cell-free fetal telomere fragments (Bredeson et al., 2014; Polettini et al., 2015a). Detection of circulating nucleic acids in maternal plasma and serum, such as cell free fetal DNA, has emerged as a predictor marker or monitoring tool for the most common and severe pregnancy complications (Phillippe, 2015). One hypothesis is that DNA telomere fragments from senescent amnion cells are shed into the amniotic fluid, and these fragments can accelerate senescence in healthy gestational tissue, as a fetal signal at term that can cause labor-associated changes (Polettini et al., 2015a). Such observation has been supported by recent *in vitro* experiments, as amniotic cells under OS produce exosomes packed with fetal telomere fragments (Sheller-Miller et al., 2017). The recognition of these molecules was speculating to be through TLR-9 that is known to trigger maternal immune cells activation in response to placenta-derived DNA (Hahn et al., 2014). However, no difference in TLR-9 expression was observed in amnion cells treated with telomere fragments compared to controls (Polettini et al., 2015a). Thus, further experiments are needed to address the specific mechanism by which telomere fragments activate intracellular signaling in fetal and maternal cells.

Regarding methylation, chorionic villi, maternal decidua, fetal membranes, and embryonic tissues have a unique DNAm setting (Robinson and Price, 2015). Particular alterations in DNAm signatures were observed in placentas and fetal membranes with acute chorioamnionitis (Konwar et al., 2018). Moreover, many investigators have described cord blood DNAm related to prematurity and inflammation (Liu et al., 2013; de Goede et al., 2017), but data on telomere-associated methylation are scarce. Wilson et al. (2016) found a correlation between shorter TL and decreased DNAm in genes associated with the telomerase regulation in placentas. The authors have described that almost 20% of the probes within TERT gene showed significant alterations in DNAm associated with TL, but they discuss epigenetic regulation of the TERT is complex, and such changes should be observed throughout the entire TERT gene region in order to elucidate the biological relevance of DNA methylation in this region. In fetal membranes, epigenetic modifications have been described, such as non-coding RNA (lncRNA) that has also been linked to pPROM (Luo et al., 2013). However, as our knowledge, there are yet no studies that have demonstrated telomere-related methylation in fetal membranes and the possible association with parturition and gestational outcomes.

Additionally, low levels of telomerase activity have been associated with TL reduction in the placentas of babies with delayed fetal development in term pregnancies, attributed to accelerated telomere DNA loss and cellular senescence (Davy et al., 2009). Fetal membranes, in particular, maintain characteristics of pluripotent cells; therefore, it would be expected to find telomerase activity in such tissues (Zhou et al., 2013). However, potency, cellular transition capability, and migratory potential are lost in fetal membranes as gestation progresses and/or in response to OS-inducing factors (Richardson and Menon, 2018), and low telomerase activity was detected in fetal membranes regardless term or PTL (Colatto et al., 2020). Accordingly, amniotic fluid derived cells, including amniotic cells, also lack telomerase activity (Chen et al., 2013). Alternatively, ALT can be activated when telomerase is suppressed; however, to date, ALT activity has not been investigated in gestational tissues. ALT regulation is influenced by telomeric repeat-containing RNA (TERRA), a lncRNA, which works as a telomerase-telomere binding inhibitor. In turn, TERRA expression is directly controlled by DNA methylation at the CpG rich gene promoters (Nabetani and Ishikawa, 2011; Coluzzi et al., 2017). In placentas, Novakovic et al. (2016) have demonstrated higher TERRA expression compared to matched somatic cells from cord blood, which was correlated to very low levels of hTERT in first trimester cytotrophoblasts. These data suggest that additional pathways might be involved in TL regulation other than telomerase activity in fetal membranes, but this requires further investigation.

## Discussion

A vast literature demonstrates that telomere-dependent replicative senescence in placental and fetal membranes is involved with parturition and gestational adversities. We have summarized that OS, inflammation, methylation, telomerase, and ALT are the main described mechanisms in telomere biology in fetal membranes to date (Figure 1C). Diverse DNA methylation are detectable for most cancer-CpG sites beginning 4 years of pre-diagnosis (Joyce et al., 2018); thus, similarly, the understanding of telomere biology, shortening of TL mechanisms, and methylation regulation in fetal membranes might provide early evidences during gestational periods in relation to telomere alterations and the propensity for pregnancy outcomes. Moreover, telomerase and ALT regulation are not fully understood in fetal membranes, which reinforces that further investigation is needed regarding telomere dysfunction in fetal membranes.

The mean TL set during intra uterine development likely has an impact on later extrauterine life, as variation in prenatal exposures and maternal states and conditions may impact fetal developmental trajectories. Subsequently, fetal or developmental programming may influence an individual’s susceptibility or propensity for disease in later life (Entringer et al., 2018). A recent systematic review demonstrated that maternal factors such as age, exposure to chemicals (e.g., smoking), and maternal stress during pregnancy and nutritional and sleep disorders are related to the stimulation of telomeres in fetal cells (Whiteman et al., 2017). Also, it has been documented that if TL is reduced in the newborn, the susceptibility to the development of chronic diseases in adulthood is increased (Entringer et al., 2012). Thus, in terms of fetal programming of the telomere system, the maternal-placental-fetal immune activation, characterized by the increased expression of pro-inflammatory cytokines in response to various adverse conditions during pregnancy, may have the potential to impact fetal TL. Although the maternal and the environmental exposure during the intrauterine period is correlated to the postnatal period, as well as with the outcomes of newborn infants, there is lack of such relation in fetal membranes studies and more studies are required to understand whether telomeres dysfunction in fetal membranes has a direct effect on fetal programing (Figure 1D).

Unfortunately, the ability to mediate a delay in parturition during pregnancy complications is limited, and the development of successful effective interventions requires a more complete understanding of the molecular mechanisms that trigger parturition, including telomere signals, which may vary throughout placental tissues.

## Author Contributions

JP and MGS have equally contributed to this review and approved the submitted version.

### Conflict of Interest

The authors declare that the research was conducted in the absence of any commercial or financial relationships that could be construed as a potential conflict of interest.

## References

[ref1] Adams P. D. Ivanov A. Pawlikowski J. Manoharan I. Tuyn J. V. Nelson D. M.. (2013). Lysosome-mediated processing of chromatin in senescence. J. Cell Biol. 202, 129–143. 10.1083/jcb.201212110, PMID: 23816621 PMC3704985

[ref2] Arias-Sosa L. A. (2018). Understanding the role of telomere dynamics in normal and dysfunctional human reproduction. Reprod. Sci. 26, 6–17. 10.1177/1933719118804409, PMID: 30296917

[ref3] Bandaria J. N. Qin P. Berk V. Chu S. Yildiz A. (2016). Shelterin protects chromosome ends by compacting telomeric chromatin. Cell 164, 735–746. 10.1016/j.cell.2016.01.036, PMID: 26871633 PMC4762449

[ref4] Behnia F. Sheller S. Menon R. (2016). Mechanistic differences leading to infectious and sterile inflammation. Am. J. Reprod. Immunol. 75, 505–518. 10.1111/aji.12496, PMID: 26840942

[ref5] Behnia F. Taylor B. D. B. Woodson M. Hawkins H. Kacerovsky M. Fortunato S. J. S.. (2015). Chorioamniotic membrane senescence: a signal for parturition? Am. J. Obstet. Gynecol. 213, 359.e1–359.e16. 10.1016/j.ajog.2015.05.041, PMID: 26025293

[ref6] Bekaert S. De Meyer T. Van Oostveldt P. (2005). Telomere attrition as ageing biomarker. Anticancer Res. 25, 3011–3021. PMID: 16080560

[ref7] Bell C. G. Lowe R. Adams P. D. Baccarelli A. A. Beck S. Bell J. T.. (2019). DNA methylation aging clocks: challenges and recommendations. Genome Biol. 20:249. 10.1186/s13059-019-1824-y, PMID: 31767039 PMC6876109

[ref8] Biron-Shental T. Sukenik-Halevy R. Sharon Y. Goldberg-Bittman L. (2010). Short telomeres may play a role in placental dysfunction in preeclampsia and intrauterine growth restriction. Am. J. Obstet. Gynecol. 202, 381.e1–381.e7. 10.1016/j.ajog.2010.01.036, PMID: 20350645

[ref9] Biron-Shental T. Sukenik-Halevy R. Sharon Y. Laish I. Fejgin M. D. Amiel A. (2014). Telomere shortening in intra uterine growth restriction placentas. Early Hum. Dev. 90, 465–469. 10.1016/j.earlhumdev.2014.06.003, PMID: 25010904

[ref10] Blackburn E. H. (1991). Structure and function of telomeres. Nature 350, 569–573. 10.1038/350569a0, PMID: 1708110

[ref11] Blackburn E. (2001). Switching and signaling at the telomere. Cell 106, 661–673. 10.1016/s0092-8674(01)00492-5, PMID: 11572773

[ref12] Blackburn E. H. Epel E. S. Lin J. (2015). Human telomere biology: a contributory and interactive factor in aging, disease risks, and protection. Science 350, 1193–1198. 10.1126/science.aab3389, PMID: 26785477

[ref13] Bredeson S. Papaconstantinou J. Deford J. H. Kechichian T. Syed T. A. Saade G. R.. (2014). HMGB1 promotes a p38MAPK associated non-infectious inflammatory response pathway in human fetal membranes. PLoS One 9:e113799. 10.1371/journal.pone.0113799, PMID: 25469638 PMC4254744

[ref14] Burton D. G. A. Krizhanovsky V. (2014). Physiological and pathological consequences of cellular senescence. Cell. Mol. Life Sci. 71, 4373–4386. 10.1007/s00018-014-1691-3, PMID: 25080110 PMC4207941

[ref15] Buxton J. L. Suderman M. Pappas J. J. Borghol N. McArdle W. Blakemore A. I. F.. (2014). Human leukocyte telomere length is associated with DNA methylation levels in multiple subtelomeric and imprinted loci. Sci. Rep. 4:4954. 10.1038/srep04954, PMID: 24828261 PMC4344300

[ref16] Campisi J. D’Adda Di Fagagna F. (2007). Cellular senescence: when bad things happen to good cells. Nat. Rev. Mol. Cell Biol. 8, 729–740. 10.1038/nrm2233, PMID: 17667954

[ref17] Casavant S. G. Cong X. Moore J. Starkweather A. (2019). Associations between preterm infant stress, epigenetic alteration, telomere length and neurodevelopmental outcomes: a systematic review. Early Hum. Dev. 131, 63–74. 10.1016/j.earlhumdev.2019.03.003, PMID: 30870624

[ref18] Chen Z. Jadhav A. Wang F. Perle M. Basch R. Young B. K. (2013). Senescence and longevity in amniotic fluid derived cells. Stem Cell Discov. 3, 47–55. 10.4236/scd.2013.31008

[ref19] Coburn S. B. Graubard B. I. Trabert B. McGlynn K. A. Cook M. B. (2018). Associations between circulating sex steroid hormones and leukocyte telomere length in men in the National Health and Nutrition Examination Survey. Andrology 6, 542–546. 10.1111/andr.12494, PMID: 29752772 PMC6105427

[ref20] Colatto B. N. Souza I. F. De Schinke L. A. A. Noda-Nicolau N. M. Silva M. G. Morceli G.. (2020). Telomere length and telomerase activity in foetal membranes from term and spontaneous preterm births. Reprod. Sci. 27, 411–417. 10.1007/s43032-019-00054-z, PMID: 32046424

[ref21] Coluzzi E. Buonsante R. Leone S. Asmar A. J. Miller K. L. Cimini D.. (2017). Transient ALT activation protects human primary cells from chromosome instability induced by low chronic oxidative stress. Sci. Rep. 7:43309. 10.1038/srep43309, PMID: 28240303 PMC5327399

[ref22] Cong Y. S. Bacchetti S. (2000). Histone deacetylation is involved in the transcriptional repression of hTERT in normal human cells. J. Biol. Chem. 275, 35665–35668. 10.1074/jbc.C000637200, PMID: 10986277

[ref23] Cox L. S. Redman C. (2017). The role of cellular senescence in ageing of the placenta. Placenta 52, 139–145. 10.1016/j.placenta.2017.01.116, PMID: 28131318

[ref24] Davalos A. R. Coppe J. -P. Campisi J. Desprez P. -Y. (2010). Senescent cells as a source of inflammatory factors for tumor progression. Cancer Metastasis Rev. 29, 273–283. 10.1007/s10555-010-9220-9, PMID: 20390322 PMC2865636

[ref25] Davy P. Nagata M. Bullard P. Fogelson N. S. Allsopp R. (2009). Fetal growth restriction is associated with accelerated telomere shortening and increased expression of cell senescence markers in the placenta. Placenta 30, 539–542. 10.1016/j.placenta.2009.03.005, PMID: 19359039 PMC2692289

[ref26] de Goede O. M. Lavoie P. M. Robinson W. P. (2017). Cord blood hematopoietic cells from preterm infants display altered DNA methylation patterns. Clin. Epigenetics 9:39. 10.1186/s13148-017-0339-1, PMID: 28428831 PMC5397745

[ref27] De Vitis M. Berardinelli F. Sgura A. (2018). Telomere length maintenance in cancer: at the crossroad between telomerase and alternative lengthening of telomeres (ALT). Int. J. Mol. Sci. 19:606. 10.3390/ijms19020606, PMID: 29463031 PMC5855828

[ref28] Dutta E. H. Behnia F. Boldogh I. Saade G. R. Menon R. Taylor B. D.. (2016). Oxidative stress damage-associated molecular signaling pathways differentiate spontaneous preterm birth and preterm premature rupture of the membranes. Mol. Hum. Reprod. 22, 143–157. 10.1093/molehr/gav074, PMID: 26690900

[ref29] Entringer S. Buss C. Wadhwa P. (2012). Prenatal stress, telomere biology, and fetal programming of health and disease risk. Sci. Signal. 5:pt12. 10.1126/scisignal.2003580, PMID: 23112344

[ref30] Entringer S. de Punder K. Buss C. Wadhwa P. D. (2018). The fetal programming of telomere biology hypothesis: an update. Philos. Trans. R. Soc. B Biol. Sci. 373:20170151. 10.1098/rstb.2017.0151, PMID: 29335381 PMC5784074

[ref31] Evans M. D. Cooke M. S. (2007). Oxidative damage to nucleic acids. 1st Edn. Austin, TX: Landes Bioscience.

[ref32] Fathi E. Charoudeh H. N. Sanaat Z. Farahzadi R. (2019). Telomere shortening as a hallmark of stem cell senescence. Stem Cell Investig. 6:7. 10.21037/sci.2019.02.04, PMID: 31019963 PMC6458335

[ref33] Ferrari F. Facchinetti F. Saade G. Menon R. (2016). Placental telomere shortening in stillbirth: a sign of premature senescence? J. Matern. Fetal Med. 29, 1283–1288. 10.3109/14767058.2015.1046045, PMID: 26004986

[ref34] Freund A. Orjalo A. V. Desprez P. -Y. Campisi J. (2010). Inflammatory networks during cellular senescence: causes and consequences. Trends Mol. Med. 16, 238–246. 10.1016/j.molmed.2010.03.003, PMID: 20444648 PMC2879478

[ref35] Gielen M. Hageman G. Pachen D. Derom C. Vlietinck R. Zeegers M. P. (2014). Placental telomere length decreases with gestational age and is influenced by parity: a study of third trimester live-born twins. Placenta 35, 791–796. 10.1016/j.placenta.2014.05.010, PMID: 25096951

[ref36] Gomez-Lopez N. Romero R. Plazyo O. Schwenkel G. Garcia-flores V. Unkel R.. (2017). Preterm labor in the absence of acute histologic chorioamnionitis is characterized by cellular senescence of the chorioamniotic membranes. Am. J. Obstet. Gynecol. 217, 592.e1–592.e17. 10.1016/j.ajog.2017.08.008, PMID: 28847437 PMC5800423

[ref37] GuD.LiJ.LittleJ.LiH.ZhangX. (2020). Associations between serum sex hormone concentrations and telomere length among U.S. adults, 1999-2002. J. Nutr. Health Aging 24, 48–54. 10.1007/s12603-019-1291-x, PMID: 31886808

[ref38] Hahn S. Giaglis S. Buser A. Hoesli I. Lapaire O. Hasler P. (2014). Cell-free nucleic acids in (maternal) blood: any relevance to (reproductive) immunologists? J. Reprod. Immunol. 104-105, 26–31. 10.1016/j.jri.2014.03.007, PMID: 24815811

[ref39] Hayflick L. (1965). The limited in vitro lifetime of human diploid cell strains. Exp. Cell Res. 37, 614–636. 10.1016/0014-4827(65)90211-9, PMID: 14315085

[ref40] Horvath S. (2013). DNA methylation age of human tissues and cell types. Genome Biol. 14:R115. 10.1186/gb-2013-14-10-r115, PMID: 24138928 PMC4015143

[ref41] Howcroft T. K. Campisi J. Louis G. B. Smith M. T. Wise B. Wyss-Coray T. (2013). The role of inflammation in age-related disease. Aging 5, 84–93. 10.18632/aging.100531 23474627 PMC3616233

[ref42] Hu H. Li B. Duan S. (2019). The alteration of subtelomeric DNA methylation in aging-related diseases. Front. Genet. 10:697. 10.3389/fgene.2018.00697, PMID: 30687384 PMC6333653

[ref43] Hua R. Pease J. E. Cheng W. Sooranna S. R. Viney J. M. Nelson S. M.. (2013). Human labour is associated with a decline in myometrial chemokine receptor expression: the role of prostaglandins, oxytocin and cytokines. Am. J. Reprod. Immunol. 69, 21–32. 10.1111/aji.12025, PMID: 23043391

[ref44] Iwasa H. Han J. Ishikawa F. (2003). Mitogen-activated protein kinase p38 defines the common senescence-signalling pathway. Genes Cells 8, 131–144. 10.1046/j.1365-2443.2003.00620.x, PMID: 12581156

[ref45] Jose S. S. Bendickova K. Kepak T. Krenova Z. Fric J. (2017). Chronic inflammation in immune aging: role of pattern recognition receptor crosstalk with the telomere complex? Front. Immunol. 8:1078. 10.3389/fimmu.2017.01078, PMID: 28928745 PMC5591428

[ref46] Joyce B. T. Zheng Y. Nannini D. Zhang Z. Liu L. Gao T.. (2018). DNA methylation of telomere-related genes and cancer risk. Cancer Prev. Res. 11, 511–522. 10.1016/j.physbeh.2017.03.040, PMID: 29895583 PMC6800137

[ref47] Kamel R. M. (2010). The onset of human parturition. Arch. Gynecol. Obstet. 281, 975–982. 10.1007/s00404-010-1365-9, PMID: 20127346

[ref48] Kang Y. Zhang H. Zhao Y. Wang Y. Wang W. He Y.. (2018). Telomere dysfunction disturbs macrophage mitochondrial metabolism and the NLRP3 inflammasome through the PGC-1α/TNFAIP3 axis. Cell Rep. 22, 3493–3506. 10.1016/j.celrep.2018.02.071, PMID: 29590618

[ref49] Karimi B. Nabizadeh R. Yunesian M. (2020). Association between leukocyte telomere length and serum concentrations of PCBs and organochlorine pesticides. Arch. Environ. Contam. Toxicol. 79, 122–130. 10.1007/s00244-020-00732-z, PMID: 32300848

[ref50] Kawanishi S. Oikawa S. (2004). Mechanism of telomere shortening by oxidative stress. Ann. N. Y. Acad. Sci. 1019, 278–284. 10.1196/annals.1297.047, PMID: 15247029

[ref51] Konwar C. Price E. M. Wang L. Q. Wilson S. L. Terry J. Robinson W. P. (2018). DNA methylation profiling of acute chorioamnionitis-associated placentas and fetal membranes: insights into epigenetic variation in spontaneous preterm births. Epigenetics Chromatin 11:63. 10.1186/s13072-018-0234-9, PMID: 30373633 PMC6205793

[ref52] Lappas M. Riley C. Lim R. Barker G. Rice G. E. Menon R.. (2011). MAPK and AP-1 proteins are increased in term pre-labour fetal membranes overlying the cervix: regulation of enzymes involved in the degradation of fetal membranes. Placenta 32, 1016–1025. 10.1016/j.placenta.2011.09.011, PMID: 21963187

[ref53] Liu Y. Hoyo C. Murphy S. Huang Z. Overcash F. Thompson J.. (2013). DNA methylation at imprint regulatory regions in preterm birth and infection. Am. J. Obstet. Gynecol. 208, 395.e1–395.e7. 10.1016/j.ajog.2013.02.006, PMID: 23477525 PMC3838789

[ref54] Lu W. Zhang Y. Liu D. Songyang Z. Wan M. (2013). Telomeres-structure, function, and regulation. Exp. Cell Res. 319, 133–141. 10.1016/j.yexcr.2012.09.005, PMID: 23006819 PMC4051234

[ref55] Luo X. Shi Q. Gu Y. Pan J. Hua M. Liu M.. (2013). LncRNA pathway involved in premature preterm rupture of membrane (PPROM): an epigenomic approach to study the pathogenesis of reproductive disorders. PLoS One 8:e79897. 10.1371/journal.pone.0079897, PMID: 24312190 PMC3842261

[ref56] Martin L. F. Moço N. P. de Lima M. D. Polettini J. Miot H. A. Corrêa C. R.. (2017). Histologic chorioamnionitis does not modulate the oxidative stress and antioxidant status in pregnancies complicated by spontaneous preterm delivery. BMC Pregnancy Childbirth 17:376. 10.1186/s12884-017-1549-4, PMID: 29132320 PMC5684743

[ref57] Menon R. (2016). Human fetal membranes at term: dead tissue or signalers of parturition? Placenta 44, 1–5. 10.1016/j.placenta.2016.05.013, PMID: 27452431 PMC5375105

[ref58] Menon R. Boldogh I. Hawkins H. K. Woodson M. Polettini J. Syed T. A.. (2014a). Histological evidence of oxidative stress and premature senescence in preterm premature rupture of the human fetal membranes recapitulated in vitro. Am. J. Pathol. 184, 1740–1751. 10.1016/j.ajpath.2014.02.011, PMID: 24832021

[ref59] Menon R. Bonney E. A. Condon J. Mesiano S. Taylor R. N. (2016). Novel concepts on pregnancy clocks and alarms: redundancy and synergy in human parturition. Hum. Reprod. Update 22, 535–560. 10.1093/humupd/dmw022, PMID: 27363410 PMC5001499

[ref60] Menon R. Dixon C. L. Sheller-Miller S. Fortunato S. J. Saade G. R. Palma C.. (2019). Quantitative proteomics by SWATH-MS of maternal plasma exosomes determine pathways associated with term and preterm birth. Endocrinology 160, 639–650. 10.1210/en.2018-00820, PMID: 30668697 PMC6388657

[ref61] Menon R. Polettini J. Syed T. A. Saade G. R. Boldogh I. (2014b). Expression of 8-oxoguanine glycosylase in human fetal membranes. Am. J. Reprod. Immunol. 72, 75–84. 10.1111/aji.12220, PMID: 24589083

[ref62] Menon R. Richardson L. S. (2017). Preterm prelabor rupture of the membranes: a disease of the fetal membranes. Semin. Perinatol. 41, 409–419. 10.1053/j.semperi.2017.07.012, PMID: 28807394 PMC5659934

[ref63] Menon R. Yu J. Basanta-Henry P. Brou L. Berga S. L. Stephen J.. (2012). Short fetal leukocyte telomere length and preterm prelabor rupture of the membranes. PLoS One 7:e31136. 10.1371/journal.pone.0031136, PMID: 22348044 PMC3278428

[ref64] Mitro S. D. Birnbaum L. S. Needham B. L. Zota A. R. (2016). Cross-sectional associations between exposure to persistent organic pollutants and leukocyte telomere length among U.S. adults in NHANES, 2001–2002. Environ. Health Perspect. 124, 651–658. 10.1289/ehp.1510187, PMID: 26452299 PMC4858394

[ref65] Nabetani A. Ishikawa F. (2011). Alternative lengthening of telomeres pathway: recombination-mediated telomere maintenance mechanism in human cells. J. Biochem. 149, 5–14. 10.1093/jb/mvq119, PMID: 20937668

[ref66] Novakovic B. Napier C. E. Vryer R. Dimitriadis E. Manuelpillai U. Sharkey A.. (2016). DNA methylation mediated up-regulation of TERRA non-coding RNA is coincident with elongated telomeres in the human placenta. Mol. Hum. Reprod. 22, 791–799. 10.1093/molehr/gaw053, PMID: 27604461

[ref67] O’Sullivan R. J. Kubicek S. Schreiber S. L. Karlseder J. (2010). Reduced histone biosynthesis and chromatin changes arising from a damage signal at telomeres. Nat. Struct. Mol. Biol. 17, 1218–1225. 10.1038/nsmb.1897, PMID: 20890289 PMC2951278

[ref68] Parry S. Strauss J. F. (1998). Premature rupture of the fetal membranes. Mech. Dis. 338, 663–670. 10.1056/NEJM199803053381006 9486996

[ref69] Paules C. Dantas A. P. Miranda J. Crovetto F. Eixarch E. Rodriguez-Sureda V.. (2019). Premature placental aging in term small-for-gestational-age and growth-restricted fetuses. Ultrasound Obstet. Gynecol. 53, 615–622. 10.1002/uog.20103, PMID: 30125412

[ref70] Phillippe M. (2015). Cell-free fetal DNA, telomeres, and the spontaneous onset of parturition. Reprod. Sci. 22, 1186–1201. 10.1177/1933719115592714, PMID: 26134037

[ref71] Phillippe M. Sawyer M. R. Edelson P. K. (2019). The telomere gestational clock: increasing short telomeres at term in the mouse. Am. J. Obstet. Gynecol. 220, 496.e1–496.e8. 10.1016/j.ajog.2019.01.218, PMID: 30690015

[ref72] Pickett H. A. Reddel R. R. (2012). The role of telomere trimming in normal telomere length dynamics. Cell Cycle 11, 1309–1315. 10.4161/cc.19632, PMID: 22421147

[ref73] Pineda-Pampliega J. Herrera-Dueñas A. Mulder E. Aguirre J. I. Höfle U. Verhulst S. (2020). Antioxidant supplementation slows telomere shortening in free-living white stork chicks. Proc. Biol. Soc. 287, 20191917. 10.1098/rspb.2019.1917, PMID: 31937223 PMC7003462

[ref74] Polettini J. Behnia F. Taylor B. D. Saade G. R. Taylor R. N. Menon R. (2015a). Telomere fragment induced amnion cell senescence: a contributor to parturition? PLoS One 10:e0137188. 10.1371/journal.pone.0137188, PMID: 26397719 PMC4580414

[ref75] Polettini J. Dutta E. H. Behnia F. Saade G. R. Torloni M. R. Menon R. (2015b). Aging of intrauterine tissues in spontaneous preterm birth and preterm premature rupture of the membranes: a systematic review of the literature. Placenta 36, 969–973. 10.1016/j.placenta.2015.05.003, PMID: 26004735

[ref76] Polettini J. Richardson L. S. Menon R. (2018). Oxidative stress induces senescence and sterile inflammation in murine amniotic cavity. Placenta 63, 26–31. 10.1016/j.placenta.2018.01.009, PMID: 29486853 PMC5833301

[ref77] Richardson L. Menon R. (2018). Proliferative, migratory, and transition properties reveal metastate of human amnion cells. Am. J. Pathol. 188, 2004–2015. 10.1016/j.ajpath.2018.05.019, PMID: 29981743 PMC6119821

[ref78] Robinson W. P. Price E. M. (2015). The human placental methylome. Cold Spring Harb. Perspect. Med. 5:a023044. 10.1101/cshperspect.a023044, PMID: 25722473 PMC4448590

[ref79] Rodier F. Campisi J. (2011). Four faces of cellular senescence. J. Cell Biol. 192, 547–556. 10.1083/jcb.201009094, PMID: 21321098 PMC3044123

[ref80] Romero R. Espinoza J. Kusanovic J. P. Gotsch F. Hassan S. Erez O.. (2006). The preterm parturition syndrome. BJOG 113, 17–42. 10.1111/j.1471-0528.2006.01120.x, PMID: 17206962 PMC7062298

[ref81] Romero R. Xu Y. Plazyo O. Chaemsaithong P. Chaiworapongsa T. Unkel R.. (2018). A role for the inflammasome in spontaneous labor at term. Am. J. Reprod. Immunol. 79:e12440. 10.1111/aji.12440.A, PMID: 26952361 PMC5016201

[ref83] Rosenquist T. A. Zharkov D. O. Grollman A. P. (1997). Cloning and characterization of a mammalian 8-oxoguanine DNA glycosylase. Proc. Natl. Acad. Sci. U. S. A. 94, 7429–7434. 10.1073/pnas.94.14.7429, PMID: 9207108 PMC23838

[ref84] Rossiello F. Herbig U. Longhese M. P. Fumagalli M. d’Adda di Fagagna F. (2014). Irreparable telomeric DNA damage and persistent DDR signalling as a shared causative mechanism of cellular senescence and ageing. Curr. Opin. Genet. Dev. 26, 89–95. 10.1016/j.gde.2014.06.009, PMID: 25104620 PMC4217147

[ref85] Rosso P. (1976). Placenta as an aging organ. Curr. Concepts Nutr. 4, 23–41. PMID: 1261303

[ref86] Rubtsova M. P. Vasilkova D. P. Malyavko А. N. Naraikina Y. V. Zvereva M. I. Dontsova О. А. (2012). Telomere lengthening and other functions of telomerase. Acta Nat. 4, 44–61. 10.32607/actanaturae.10630, PMID: 22872811 PMC3408703

[ref87] Salminen A. Kauppinen A. Kaarniranta K. (2012). Emerging role of NF-κB signaling in the induction of senescence-associated secretory phenotype (SASP). Cell. Signal. 24, 835–845. 10.1016/j.cellsig.2011.12.006, PMID: 22182507

[ref88] Sanchez M. Hoang S. Kannengiesser C. Potier L. Hadjadj S. Marre M.. (2020). Leukocyte telomere length, DNA oxidation, and risk of lower-extremity amputation in patients with long-standing type 1 diabetes. Diabetes Care 43, 828–834. 10.2337/dc19-0973, PMID: 31988064

[ref89] Sheller-Miller S. Urrabaz-Garza R. Saade G. Menon R. (2017). Damage-associated molecular pattern markers HMGB1 and cell-free fetal telomere fragments in oxidative-stressed amnion epithelial cell-derived exosomes. J. Reprod. Immunol. 123, 3–11. 10.1007/s10995-015-1800-4, PMID: 28858636 PMC5632595

[ref90] Shin J. Y. Choi Y. Y. Jeon H. S. Hwang J. H. Kim S. A. Kang J. H.. (2010). Low-dose persistent organic pollutants increased telomere length in peripheral leukocytes of healthy Koreans. Mutagenesis 25, 511–516. 10.1093/mutage/geq035, PMID: 20616147

[ref91] Smith R. Maiti K. Aitken R. J. (2013). Unexplained antepartum stillbirth: a consequence of placental aging? Placenta 34, 310–313. 10.1016/j.placenta.2013.01.015, PMID: 23452441

[ref92] Stewart S. A. Ben-Porath I. Carey V. J. O’Connor B. F. Hahn W. C. Weinberg R. A. (2003). Erosion of the telomeric single-strand overhang at replicative senescence. Nat. Genet. 33, 492–496. 10.1038/ng1127, PMID: 12652299

[ref93] Sutanto S. S. I. McLennan S. V. Keech A. C. Twigg S. M. (2019). Shortening of telomere length by metabolic factors in diabetes: protective effects of fenofibrate. J. Cell Commun. Signal. 13, 523–530. 10.1007/s12079-019-00521-x, PMID: 31203557 PMC6946782

[ref94] Tan R. Lan L. (2017). “Induction of site-specific oxidative damage at telomeres by killerred-fused shelretin proteins” in Telomeres and telomerase: Methods and protocols, methods in molecular biology. ed. Songyang Z. (Berlin/Heidelberg, Germany: Springer Science+Business Media LLC), 139–146. 10.1007/978-1-4939-6892-3_14 28324506

[ref95] Toubiana S. Selig S. (2020). Human subtelomeric DNA methylation: regulation and roles in telomere function. Curr. Opin. Genet. Dev. 60, 9–16. 10.1016/j.gde.2020.02.004, PMID: 32109830

[ref96] Ventura M. T. Casciaro M. Gangemi S. Buquicchio R. (2017). Immunosenescence in aging: between immune cells depletion and cytokines up-regulation. Clin. Mol. Allergy 15:21. 10.1186/s12948-017-0077-0, PMID: 29259496 PMC5731094

[ref97] von Zglinicki T. (2002). Oxidative stress shortens telomeres. Trends Biochem. Sci. 27, 339–344. 10.1016/S0968-0004(02)02110-2, PMID: 12114022

[ref98] von Zglinicki T. Pilger R. Sitte N. (2000). Accumulation of single-strand breaks is the major cause of telomere shortening in human fibroblasts. Free Radic. Biol. Med. 28, 64–74. 10.1016/S0891-5849(99)00207-5, PMID: 10656292

[ref99] Whiteman V. E. Goswami A. Salihu H. M. (2017). Telomere length and fetal programming: a review of recent scientific advances. Am. J. Reprod. Immunol. 77:e12661. 10.1111/aji.12661, PMID: 28500672

[ref100] Wilson S. L. Liu Y. Robinson W. P. (2016). Placental telomere length decline with gestational age differs by sex and TERT, DNMT1, and DNMT3A DNA methylation. Placenta 48, 26–33. 10.1016/j.placenta.2016.10.001, PMID: 27871469

[ref101] Xu Z. Duc K. D. Holcman D. Teixeira M. T. (2013). The length of the shortest telomere as the major determinant of the onset of replicative senescence. Genetics 194, 847–857. 10.1534/genetics.113.152322, PMID: 23733785 PMC3730915

[ref102] Yeap B. B. Hui J. Knuiman M. W. Paul Chubb S. A. Ho K. K. Y. Flicker L.. (2020). Associations of plasma IGF1, IGFBP3 and estradiol with leucocyte telomere length, a marker of biological age, in men. Eur. J. Endocrinol. 182, 23–33. 10.1530/EJE-19-0638, PMID: 31658437

[ref103] Yeap B. B. Knuiman M. W. Divitini M. L. Hui J. Arscott G. M. Handelsman D. J.. (2016). Epidemiological and mendelian randomization studies of dihydrotestosterone and estradiol and leukocyte telomere length in men. J. Clin. Endocrinol. Metab. 101, 1299–1306. 10.1210/jc.2015-4139, PMID: 26789780

[ref104] Zhang M. Hu M. L. Huang J. J. Xia S. S. Yang Y. Dong K. (2019). Association of leukocyte telomere length with non-alcoholic fatty liver disease in patients with type 2 diabetes. Chin. Med. J. 132, 2927–2933. 10.1097/CM9.0000000000000559, PMID: 31809318 PMC6964937

[ref105] Zhou K. Koike C. Yoshida T. Okabe M. Fathy M. Kyo S.. (2013). Establishment and characterization of immortalized human amniotic epithelial cells. Cell Rep. 15, 55–67. 10.1089/cell.2012.0021, PMID: 23298399 PMC3567704

